# FitzHugh-Nagumo to model a large number of diffusive coupled neurons

**DOI:** 10.1186/1471-2202-14-S1-P53

**Published:** 2013-07-08

**Authors:** Anna Cattani, Claudio Canuto

**Affiliations:** 1 Mathematical Sciences, Polytechnic of Turin, 10129, Italy

## 

The aim of our work is to investigate the dynamics of a neural network, in which neurons, individually described by the FitzHugh-Nagumo model [1], are coupled by a generalized diffusive term. The formulation we exploit is based on the general framework of graph theory, where neurons are represented by vertices and links by edges.

With the aim of defining the connection structure among the excitable elements, the discrete Laplacian matrix plays a fundamental role. Indeed, it allows us to model the instantaneous propagation of (electric) signals between neurons, which need not be physically close to each other. This approach enables us to address three fundamental issues. Firstly, each neuron is described using the well-known FitzHugh-Nagumo model which might allow to differentiate their individual behavior. Furthermore, exploiting the Laplacian matrix, a well defined connection structure is formalized. A thoroughly explained mathematical structure allows us to formally describe several fundamental features of interactions in neural populations. Indeed, the description of not only nearest neighbor interactions and the presence of inhibitory synapses has been achieved. Several simulations are performed to graphically present how the action potential within a network evolves. In general, placing the neuron in line and stimulating one of them, two waves which carry out the connection rule arise.

**Figure 1:**
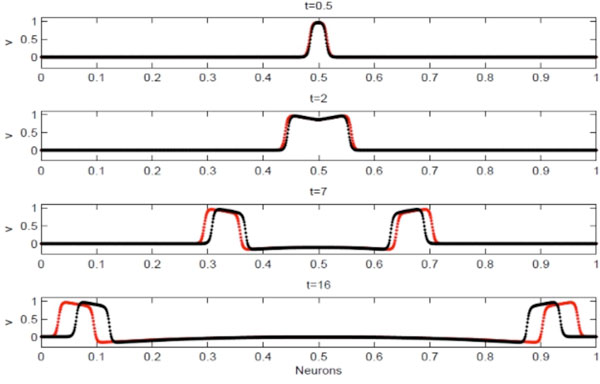
**Comparison of dynamics obtained by considering 1024 neurons**. The stimulus consists in imposing a non-null initial action potential on the central node of the set. At the end of the integration all neurons return to the quiescent state due to the physiological refractory period. Dynamics obtained by using Approach I (red dots) and Approach II (black dots) are shown. With greater number of neurons the error between the results by Approach I and II tends to zero.

While the number of neurons N increases in a bounded domain, without any changes in the model, the effect is that the two arisen waves travel slower along the whole set of neurons. Neglecting in a first approximation the time delays due to the chemical synapses, we formalize two approaches which allow us to reproduce the qualitative behavior of the action potential as N increases, i.e., waves assume the same shape and spend the same time to travel along the same length interval. Concisely, Approach I consists of rescaling the diffusion coefficient while Approach II consists of increasing, in accordance with a specific law, the number of connections per neuron. In order to make clear the graphical comprehension of the dynamics, only nearest neighbor interactions are allowed in Approach I. Furthermore, a comparison between them has been performed. An example of dynamics obtained by exploiting both approaches in one-dimension is shown (see Figure 1).

A continuum of neurons is the results of the limit process of letting N to infinity and a system of convection/reaction/diffusion partial differential equations describes the action potential and the recovery variable in the whole set of neurons.

Finally, our two approaches immediately extend to higher dimensions.
